# Parenting roles for young people with attention‐deficit/hyperactivity disorder transitioning to adult services

**DOI:** 10.1111/dmcn.15320

**Published:** 2022-06-20

**Authors:** Astrid Janssens, Sharon Blake, Helen Eke, Anna Price, Tamsin Ford

**Affiliations:** ^1^ User Perspectives and Community‐based Interventions, Department of Public Health University of Southern Denmark Odense C Denmark; ^2^ Center for Forskning Sammen med Patienter og Pårørende Odense University Hospital Denmark; ^3^ University of Exeter Medical School Exeter; ^4^ Law School University of Exeter Exeter; ^5^ Department of Psychiatry University of Cambridge Cambridge UK

## Abstract

**Aim:**

To inform transitions from child to adult health services, we explored the work and roles parents take in the care of young people with attention‐deficit/hyperactivity disorder (ADHD) aged 14 to 25 years old.

**Method:**

Using framework thematic analysis, we analysed data collected from 28 semi‐structured interviews with parents of young people with ADHD to generate a typology and triangulated it against findings from 64 interviews with young people with ADHD. The interviews were carried out as part of a three‐strand, interactive mixed‐method study.

**Results:**

An entourage typology of three parent roles was identified. Parents moved between ‘manager’ and ‘roadie’ roles as their child gradually matured. A ‘superfan’ role was identified which supported young people's positive self‐image but may impede withdrawal from the ‘manager’ role. Continued parental involvement into adulthood reflected a need to maintain the balance of resources required to maintain quality of life for the whole family.

**Interpretation:**

This is the first study to explore parental roles in the health care of young people with ADHD. Parents will vary in their capacity to fulfil the identified roles and step back their care as their children reach adulthood. The findings can inform intervention development to support families and transition between services.

**What this paper adds:**

- Parents move from a ‘manager’ to ‘roadie’ role as young people mature.
- A ‘superfan’ role supports positive self‐image and directed health care work.
- Continued involvement reflects parental responsibility to juggle wider family needs and resources.
- Parents differ in capacity to fulfil and move between these roles.

## What this paper adds

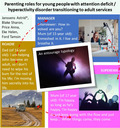

Plain language summary: https://onlinelibrary.wiley.com/doi/10.1111/dmcn.15599

**Abbreviation**
LTHC: Long‐term health condition.

Attention‐deficit/hyperactivity disorder (ADHD) is classified as a childhood‐onset neurodevelopmental disorder defined by the presence of a persistent pattern of inattention and/or hyperactivity–impulsivity that interferes with functioning or development. Individuals with ADHD often find organization and time management challenging, with associated negative outcomes in education, employment, and relationships.^1^ ADHD is frequently comorbid with other psychiatric disorders and there is an increased risk of mortality, driving accidents, divorce, and contact with criminal justice agencies, particularly if left untreated.^2^, ^3^ In DSM‐5, ADHD is included as a lifespan neurodevelopmental condition to recognize that many young people experience ongoing difficulties into adulthood.^1^ Medication‐based treatments are widely used and have proven efficacy in the short term; however, long‐term effectiveness remains uncertain.^4^ A high rate of medication discontinuation occurs around puberty despite increased severity of impairments at that age.^5^ This and low numbers of patients with ADHD who successfully transition to adult services in the UK^6^ has focused attention on what can help support continuity of care. Existing studies have suggested parents are a key component for successful transition from child to adult services, with mothers typically taking responsibility for the transition process.^7^, ^8^ Based on earlier findings from our qualitative study exploring ADHD service transition in the UK,^7^ in this study we present a typology of the roles and work parents of young people with ADHD adopt in the illness trajectory and how these change during transition to adulthood. We apply the term ‘parent’ to mean any significant other acting as a parent or carer, and ‘child’/‘young people’ to denote individuals aged between 14 and 25 years old.

Health care for young people with ADHD in the UK is provided free of charge through a public taxation funded National Health Service (NHS). However, access depends on age, severity, comorbidities, and availability of services which vary regionally and can involve long waiting lists.^7^, ^9^ Up until the age of 16 years, or 18 years if in full‐time education, support for ADHD may be provided by community paediatricians or child and adolescent mental health services. Young people are assessed as they approach the upper age limit for child services and if criteria are met for continuing care as an adult, this is typically provided by community‐based adult mental health teams. While UK health care policies stress the importance of parent involvement in planning for transition between child and adult services, adult health care services emphasize self‐management for patients.^10^, ^11^, ^12^ As such, parents' automatic right to be included in formal health care engagement typically ends when their child reaches around 18 years of age.^7^, ^13^ This fails to recognize that during ‘emerging adulthood’ young people between the age of 18 and 25 years may view themselves as neither adolescents nor adults but something in between,^14^ nor that parents typically continue to provide practical, financial, and emotional support past the legal age of majority. Parents of individuals with cognitive disorders in particular will continue to be an important resource for their child in emerging adulthood as they face simultaneous adjustments such as participating in full‐time work, carrying out daily tasks, and managing social relationships.^13^

Parents often report feeling helpless and excluded whilst at the same time taken for granted by health services.^15^ Parents have also acknowledged how it can be a struggle to balance protection from poor outcomes with passing on responsibilities to their children, in particular, coordinating care and clinical decision‐making.^16^, ^17^ Systematic reviews have reported parents can experience anxiety and have trouble in letting go of health care responsibilities at transition.^18^, ^19^ Heath et al.^20^ suggested that health care professionals need to provide clarification to parents as to how they can adjust their roles to promote autonomy and reduce the negative impact on both their own well‐being and their child's transition to self‐care. They suggest that the issues parents experience during health care transition are generic. A small in‐depth study indicated that because of the associated risks of ADHD (impulsivity, disorganization), parents considered that the level of ‘protective vigilance’ required to safeguard their child with ADHD was significantly different to that of a child without ADHD.^21^ So, what roles do parents of young people with ADHD currently adopt in the illness trajectory? How does this change as their children enter adulthood?

While the contribution of parents in the care of those with chronic conditions has been recognized, little attention has been paid to developing an understanding of the type of work undertaken within social networks.^22^ Our approach in this study was informed by Corbin and Strauss' theoretical work exploring how chronic illnesses are managed at home.^23^ Expanding their theory beyond spousal care, we were interested in the ‘work’ undertaken by parents in the care of young people with ADHD at a time when biographical work (move from dependence to interdependence) requires a reorganization of responsibilities. What tasks were involved, who did what, and what issues were encountered in relation to illness‐related work (e.g. treating symptoms, crisis management, and interactions with health care services) and everyday‐life work (e.g. housekeeping, occupational work)?

Existing research suggests managing children's long‐term health conditions (LTHCs) requires an ability to cope with uncertainty and negotiation between parents to provide practical and emotional support, as well as accommodate the needs of other children and paid employment.^24^ Two systematic reviews of studies exploring the experience of parenting a child with LTHCs into adulthood report that families try to balance the child's developmental need for independence against potential consequences of poor self‐management by gradually progressing from parental management towards self‐care.^12^, ^20^ They suggest parents incrementally upskill their children in skills such as medication management, but other skills such as decision‐making and liaising with services need to be acquired quickly on transfer to adult services.

Woodgate et al.^25^ found seven roles which parents take in caring for children with LTHCs and suggest that in transition between child and adult health systems, the ‘teacher’ and ‘advocate’ roles are particularly necessary to ensure support is in place. They identified protection of young people's psychosocial well‐being was of most concern for parents during transition. Where risk of harm, which included inadequacies in health care support and unpredictable illness trajectory, were perceived to have diminished, parents felt more ready to hand over responsibility. These studies looked across health issues, but understanding is also needed as to how these findings relate to specific conditions.

To our knowledge, this is the first study to specifically explore the role parents take in the care of young people with ADHD and how roles change as their children transition to adulthood. Initiating an understanding of the work undertaken by parents aims to contribute to theoretical development and future research as well as inform policymakers and services as to the resources required in the management of ADHD, particularly during transition to emerging adulthood.

The qualitative data drawn on herein were collected between 2016 and 2018 as part of a wider explorative mixed methods study which aimed to understand and assess the transition from child to adult health services for young people with ADHD in the UK: the CATCh‐uS study.^7^ This study consisted of three parts: (1) a surveillance study describing the transition process and those in need of transition; (2) a mapping study to describe available adult ADHD health care services in the UK; and (3) a qualitative study to understand transition experiences of those involved. Quantitative and qualitative methods were used both sequentially and in parallel. The surveillance study provided insight into the factors of optimal transition and referral destination for young people who were becoming too old for child services. This information guided the qualitative study which aimed to explore how the identified factors influencing transition through the quantitative studies compared to individual experience. Interviews were conducted with 144 individuals from seven stakeholder groups: (1) young people pretransition; (2) young people who transitioned directly to adult services; (3) young people who did not transition but later accessed adult services; (4) parents of children with ADHD (some of whom were pretransition, some posttransition, and some who did not transition); (5) paediatricians and child psychiatrists; (6) health professionals working in adult mental health services; and (7) general practitioners. We were interested in the illness‐related work undertaken by parents for this paper; therefore, we focused on the parent and young people interviews to explore: (1) What roles do parents take in the care of young people with ADHD? (2) How does this work change along the illness trajectory and particularly as their children transition into emerging adulthood?

## METHOD

### Data collection

We aimed to recruit 25 parents with a child in services and 20 to 25 young people across transition points (pre‐, post‐, or no transition). Our sample size targets were informed by previous studies on transition and wider methodological findings regarding anticipated information power relevant to explore similarities and differences.^26^ We recruited using a sampling matrix to ensure variety in location and type of service provision (with or without follow‐up adult services for ADHD), sex, and age. The sampling frames were verified against the socioeconomic characteristics for young people eligible for transition as collected via the surveillance study.^27^ Selection of participants was guided by patient comorbidity, residence of participant (with parents or elsewhere), occupation (school; higher education; employment; or not in education, employment, or training), IQ (if provided), special educational needs, and contact with criminal justice. Recruitment was continuously monitored to ensure that the sampling frame was being evenly populated. Potential young people and parent participants were identified by clinical research nurses at 10 NHS provider organizations (Trusts).^7^

Face‐to‐face, semi‐structured interviews were conducted by trained interviewers (HE, AP, AJ) who had experience of working with young people with neurodisability, at the participant's home, or at a hospital lasting on average 1 hour. We chose in‐person semi‐structured interviews as we felt this method would best allow comparison across interviews whilst still allowing participants to direct us to what was important to them. Young people were asked if they wanted to bring a confidant to the interview and some invited their mother. When young people did not know the answers to certain questions, they asked their mother (for example: when they started medication) and some mothers also contributed uninvited. If this happened frequently or at length, we respectfully reminded confidants that we were there to hear the young person's perspective.

Interim analyses were performed as data collection progressed, to inform recruitment and adjust the topic guides to further capture areas of interest. Interviews were audio‐recorded, transcribed verbatim, edited to remove identifying details, and managed using QSR International NVivo 11 software (QSR International, Burlington, MA, USA. For more details about the sample and the method, including the interview topic guides, please see the CATCh‐uS study report.^7^

### Ethics

The CATCh‐uS study and subsequent amendments received ethics committee approval from the National Research Ethics Service Committee Yorkshire and the Humber—South Yorkshire; the original protocol was approved in October 2015 (REC reference 15/YH/0426). NHS Research and Development Office approval was attained in all 10 hospital trusts.

Before the interview, the researcher discussed the study with the participant, who was then given the opportunity to ask any questions. Once they agreed to continue with the interview, they were asked to sign the consent form. Parents of young people aged 15 years or younger were asked to consent to the participation of their child, in addition to seeking assent from the young person.

### Analysis

A framework thematic analytical approach was adopted. After familiarizing with the data, framework categories were identified and a matrix created, with a focus on organizing the data in a meaningful and manageable way. This was followed by charting (summarizing the indexed data) and mapping the data to explore patterns and articulate one's own sense‐making, in the light of the research question(s).

The charting of the parent interviews revealed that they took a strong, active, and continued role in the engagement with services and treatment of their child, whether at a young age or into their early adulthood.^7^ For this study, we explored the views and experiences of parents of children with ADHD with a particular focus on how parents relate themselves to the care of their child, the range and nature of the caring roles adopted, and how this relationship evolved as their child went towards and beyond transition into adult services.^28^ From this analysis, we created a typology for the caring roles. The typology presented in this paper is grounded in the parent interview data. To explore convergence, complementarity, and dissonance with what the young people interviewed had shared in respect of their parent's involvement,^29^ relevant data from the young people interviews (with either positive or negative examples) are also presented.

## RESULTS

In total, we conducted 92 interviews. Twenty‐seven mothers and three fathers (two of whom participated in interviews held with the mother) contributed; the mean age of young people was 19 years (range 14–28 years). We interviewed 64 young people with ADHD who were in contact with health services, 20 females and 44 males, at different ages and stages of the transition process. Characteristics of the participating young people are shown in Table 1. Supporting quotes for the findings are set out in Table S1 with corresponding Roman numerals provided within the text in brackets.

**Table 1:** Interviewee age, sex, and reported comorbidity characterisitics

Interviewee	Young people, age range: years	Young people, sex: female/male	Young people, comorbidities
Young people pretransition; *n* = 21	14–17	5/16	Yes: 9 None: 11 Unknown: 1 Confirmed ASD: 8
Young people posttransition; *n* = 22	17–21	9/13	Yes: 14 None: 6 Unknown: 2 Confirmed ASD: 10
Young people re‐entering adult services; *n* = 21	19–29	6/15	Yes: 13 None: 8 Unknown: 0 Confirmed ASD: 7
Parents of child with ADHD; *n* = 28	14–28	6/22	Yes: 14 None: 12 Unknown: 2 Confirmed ASD: 8

Abbreviations: ASD, autism spectrum disorder; ADHD, attention‐deficit/hyperactivity disorder.

**Figure 1:**
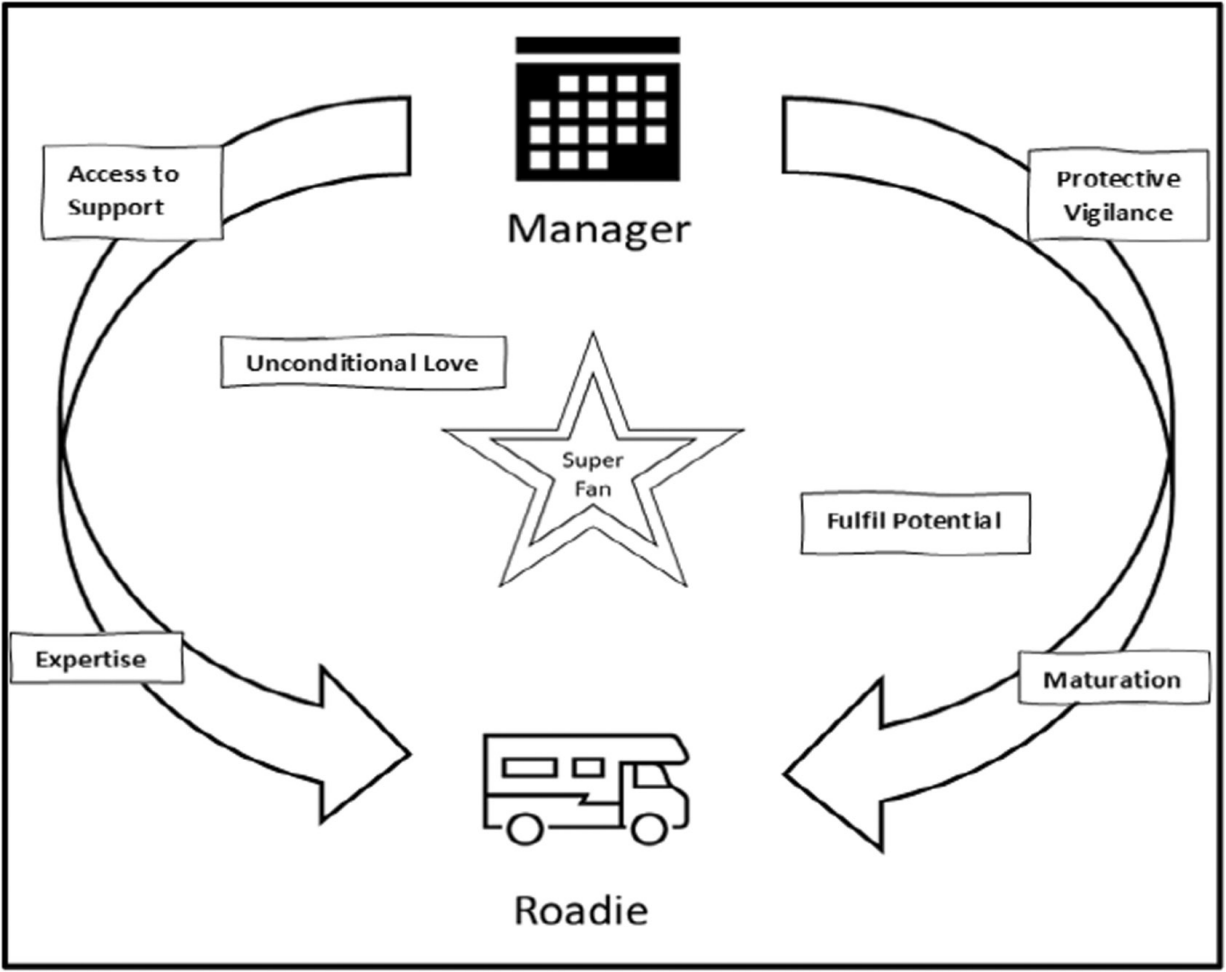
Health care engagement and treatment roles adopted by parents of young people with attention‐deficit/hyperactivity disorder.

The parent interviews suggest three roles which parents of young people with ADHD take in relation to their engagement with health services and the treatment and management of their child's ADHD. Using a rock star analogy, we developed an ‘entourage typology’ of these roles: ‘manager’, ‘roadie’, and ‘superfan’. If parents felt their child had access to support as they matured and the requisite expertise, including ability to manage risks associated with ADHD, they moved from a manager to a roadie role. Parents undertook a superfan role before, during, and after transition wherein through unconditional love they stood by their children and worked to find solutions which would support them to fulfil their potential (Figure 1).

### Parent as manager

Parents described an essential nature to their involvement in their child's engagement with health care services. They arranged appointments; transported and accompanied them; completed assessment forms with them; translated information received from clinicians into a form their children understood and repeated it over time. Parents explained that without their support, the young people would not access services (i). Some parents described strong informal support networks—both their own and their children's—where they could ask for advice and rely on support. However, for others, social exclusion reinforced perceptions of the parent's essential role (ii).

Despite reservations regarding young people's independence, the belief that parents needed to speak for their children reflected their experience of the effort required to access support. Peppered throughout the interviews were references to poorly resourced health care services. These were reasoned by the parents to explain their experiences of staff turnover, lengthy waiting lists, limited time with clinicians, and discharge processes which left families feeling concerned and unsupported. Parents described how their child's complex needs or lack of ‘severity’ did not always fit the services available. Navigating services, which frequently changed how they were accessed or how they were delivered, required patience, persistence, and skills that many people, not least young people, would find challenging (iii).

The view of essentiality regarding engagement with health care services was also reflected in parent views of their role in their child's treatment for ADHD. Parents ensured that their children were eating and maintaining personal hygiene. They made sure prescriptions were filled and helped their children learn and implement coping strategies. Parents specifically emphasized managing their children's ADHD medication. They administered medication and observed and helped make decisions regarding trials of different medications and dosages. They ensured compliance with medication plans and slow withdrawal when needed. They explained that their involvement reflected chronic disorganization linked to their child's ADHD, potential misuses of classified medications, concerns regarding side effects which they thought their children may not be able to voice, and their greater understanding of how the medication may help (iv).

Parents reflected on the time and skill required to manage their children's health care. Interlinked with their superfan role, they made sacrifices to prioritize their children's care and reported they would or had sought private health care support if NHS support was not available. Taking a managerial role meant that they were better able to juggle their children's needs and the demands of the health care system with their work, finances, and wider family well‐being (v). The expertise they had developed in accessing support and in understanding their children's ADHD and treatment also contributed to a view of their essential managerial role. All of the interviewed parents felt that their children would have ongoing needs as an adult as a result of their ADHD (vi) and beliefs were reported about the greater risks faced by young people with ADHD which justified continued protective vigilance (vii).

The interviews with pretransition and posttransition young people matched the description of the work parents took in relation to diagnosis, symptom management, and in particular medication management, and dealing with the health care system (viii). All pretransition young people and most of the posttransition young people were heavily reliant on their parent(s) to deal with services. Views were expressed that they hoped to be better able to deal with things themselves in the near future. Some of the non‐transition young people also told us that their mother or partner was their main support in management of their ADHD. While it was rare for these emerging adults to exclusively rely on parental support, they continued to recognize their input (ix).

### Parent as roadie

Parents talked about the importance of independence or a level of independence for their children as they entered adulthood. They were keen to avoid dependence, despite expressing continuing fears, particularly in relation to the young people with ADHD driving vehicles (x). Some described gradually pulling back from a managerial role to more of a roadie role in relation to health care engagement and treatment. They still transported their children to appointments but would stand back and encourage them to make decisions regarding treatment (xi). Parents wished for consistent support from health care services to prepare their children for transition and adult life. They expressed a lack of faith that clinicians understood or cared that their children might not be emotionally ready to transition to adult services or engage with health care services without their involvement (xii).

Maturation was key for parents to move from a managerial to a roadie role where they would try to take a step back. Parents felt that their children's management of their ADHD would improve with maturity. Maturity was signalled to the parents by young people demonstrating both an understanding of how ADHD affected them, and an acceptance of ADHD as a long‐term condition. This involved seeing that young people were learning to live with their ADHD and making informed decisions about treatment (xiii).

Parental support in the background is illustrative of many young people's posttransition perceptions of their parent's role in their life. They similarly report parent roles have shifted as they matured but accepted a need for continuing support (xiv).

### Parent as superfan

Parents described significant challenges their children had faced in relation to staying in school; finding suitable employment; and/or avoiding violence (towards self/others), criminality, and addiction (xv). Reflecting views that there was little understanding of ADHD in wider society, through unconditional love parents stood by their children when their ADHD symptoms were challenging, despite impacts on their own well‐being. Short‐term treatment was often focused on the school day, which meant that challenging ADHD symptoms could flare before or after school at home. They understood that their children's challenging behaviour often reflected an unmet need such as anxiety, hunger, frustration with communication, or overstimulation (xvi).

Parents were driven to see their children fulfil their potential. Diagnosis and treatment were often sought to help academic performance at school. Parents described their own aspirations for their children to live independently, to have positive relationships and successful careers. They explained how they had to adjust their aspirations as they understood the impact of their children's ADHD (xvii) but wanted to support their children with their own plans for the future, including finding a specialized interest, niche, or environment which would align with their ADHD symptoms (xviii). As superfans, they found out how their children felt and worked out how to engage with health care to best reflect what would work for their children. For example, parents reported their children as conscious of being different and embarrassed by a need to take medication. They described wanting to support their children's desire to be ‘normal’ (xix). Parents found accessing mental health services for their children unsettling and reported having to overcome feelings of shame or discrimination related to an ADHD diagnosis, plus concerns over medicating their children (xx). Being a superfan meant putting aside their individual concerns to see a fuller picture.

Pre‐ and posttransition young people gave credit to their parents' efforts for being the person they had become, acknowledging the huge role their mother played in their development and journey through services (xxi).

### Parents not taking on the illness‐related work

The interviews with non‐transition young people suggested that conflicts can impact the degree to which parents can take on a superfan, manager, or roadie role in the ADHD illness trajectory. Some shared that their parents did not help them engage with health services, or manage their condition, as they did not agree with a mental health approach for support or treatment (xxii). Others struggled with negative perceptions they felt their parents held about them or felt that they did not have a say in respect of a ‘pushy’ managerial role (xxiii).

## DISCUSSION

The findings of this study suggest an ‘entourage typology’ of three roles which parents take in the health care of young people with ADHD: manager, roadie, and superfan. Within the manager role, parents of young people with ADHD described many aspects of illness‐related work highlighted by Corbin and Strauss.^23^ They led the engagement with health care services, arranged and accompanied their children to clinician appointments, monitored effects of trial medications, and ensured compliance with treatment plans. As managers, parents also supported their children with everyday‐life work tasks which they struggled with, such as diet and personal hygiene. The manager role reflected the expertise parents had developed to understand their children's ADHD and, in keeping with existing studies, the effort required to access and sustain health care support.^16^, ^25^ In line with Corbin and Strauss,^23^ parents described the essentiality of this role in order to maintain relative equilibrium. They needed to juggle the illness‐related work in respect of their children with ADHD with their occupational work and the wider family well‐being, with the level and type of input required subject to change according to circumstances (e.g. if parent loses job or young person reacts badly to medication).^22^ Parents take a manager role to be able to prioritize the resources and effort required to respond as best as they can to inevitable changes.

As young people transition into adulthood, parents described moving from a manager role to a roadie role. Reflecting an importance attached to independence for their ‘emerging adults’, parents described pulling back from managing their children's treatment and engagement with health care services. As a roadie, they would still provide tangible support, such as collecting prescriptions or providing transport to health care appointments, but they would stand back and encourage their children to make decisions.

The superfan role supports young people throughout the illness trajectory with unconditional love, steering away from critical voices in a drive to see their children fulfil their potential. As a superfan, parents focused on the young people's well‐being and happiness, keeping strengths of their children in the foreground. This focus involves adopting a longer temporal perspective, with parents acting on the basis of transferred preferences for the long term, as well as immediate concerns. This was most apparent through the link parents made in seeking diagnosis and treatment for their children's ADHD to address behaviour in school and academic achievement. Corbin and Strauss^23^ emphasize the importance of mutuality in the familial care of individuals with LTHC. Although it is difficult to compare directly with their work because of the different dynamics in spousal relationships as opposed to parent–child relationships, mutuality arguably also appears to be a driver and goal for superfan parents. Reflecting the finding that retaining responsibility for everyday‐life tasks provide a higher value than illness‐related work for individuals with LTHCs,^22^ parents actively sought and fostered the positive contribution their children made to everyday‐life tasks. They then in turn complemented their actions by taking the lead in the illness management work.

### Parent support during transition to emerging adulthood

In line with existing literature, parents sought recognition both of their continued supporting role in emerging adulthood to reduce potential consequences of poor self‐management and gradual lessening of their involvement based on their perceptions of young people's maturation.^30^, ^31^ Parents described an interlinked ‘protective vigilance’ to safeguard their children because of increased risks associated with the impulsivity and poor organization of ADHD^21^ and a lack of availability and trust in adequate health care support which has been recognized as undermining transition to self‐care.^13^, ^16^, ^20^ They felt ADHD was a poor fit with health services which focus on episodic and crisis care and explained how a lack of severity of a recognized mental illness, or complexity of symptoms, meant that they had to continue their involvement in the management of their children's ADHD unless they had access to informal or private health care support. What this study adds to our understanding of continuing parental involvement into adulthood is the need for parents to maintain a state of relative equilibrium for a wider family network. Parents required resources to move to a roadie role and support self‐management whilst also balancing the needs of others and themselves. To give a simple example, it would be easier for parents to arrange time off work and take their children to a clinic appointment then to have to drop things at short notice to collect them should their disorganization mean they miss a bus. It is therefore not always an overestimation of risks of young people's autonomy as per Heath et al.,^20^ but a shortfall of resources which means parents have to choose which needs to try and meet and how.

The identified superfan role may also impede the move from manager to roadie. Reflecting awareness of long‐term ADHD affects and the lack of societal value afforded to ADHD attributes, parents adopted a superfan role to support their children's positive self‐image. For example, by respecting their desire to fit in and looking for ways to integrate health care management into their children's overall life projects and positive activities which aligned with their ADHD symptoms. However, feeling like you are their only cheerleader and habitually adopting a strengths‐based perspective may make it difficult to step back, or indeed realistically identify behaviour which may need to be addressed. As O'Hara et al.^32^ have suggested, parents may need support to move to a more peripheral role and we hope that the above identified barriers will help focus where that support may be needed.

Overall, the interviews with young people supported the roles and work which parents reported undertaking in the management of their children's ADHD as they transitioned into adulthood. However, compared to the young people who had experienced continuity of services, some of the non‐transition young people described conflicts which impacted the degree to which their parents fulfilled these roles. They reported parents struggling to either reconcile their negative views of ADHD treatment with continuing support to access services or balance their developed expertise and concern for poor outcomes with young people's desire for independent decision‐making and need for affirmation. In part, as some of the non‐transition young people had moved out of the family home, changes were inevitable in the work and role parents took in the management of their ADHD. However, the conflicts could also reflect the strain ADHD symptoms can put on the parent–child relationship^33^ or social class differences, as Olsvold et al.^34^ found resistance to medical understandings of ADHD were clearer in working‐class narratives.

The extra effort parents of young people with LTHCs commit to, whilst still balancing all other aspects of family life and work commitments, has led to the description of their role as intensive parenting.^25^ Not all parents have the time or skill to fulfil these roles. Parents facing material deprivation already have to deal with more contingent stress,^35^ and in the case of ADHD, the negative impact on parent health‐related quality of life has been recognized,^36^ as has the likelihood that the parent themselves may have ADHD^37^ which may each limit capacity to support the health care needs, including increasing autonomy of their children.

### Conclusion

In this study we have presented a typology of roles and work undertaken by parents of young people with ADHD in the illness trajectory and how these can change during transition to adulthood. Our findings support proposals for developmentally appropriate transition outcomes which recognize the dynamic impact environmental, psychosocial, and vocational aspects, as well as medical care have on adolescent development to better support transition to emerging adulthood.^10^, ^38^

Much work has been undertaken to explore why transition from child to adult health services fails. In this study we have offered a novel approach to better understand the relational activities in the ADHD illness trajectory and why parents continue to be involved in emerging adulthood. How a parent fulfils one of the identified roles may influence engagement with health care services and the success of transition. Restricting parental involvement and withdrawing support for the family during emerging adulthood is unlikely to help parents move to a roadie role. Future research could explore how the typology may offer insights for interventions aiming to promote understanding and support the needs of maturing young people with ADHD and their families.

The presented typology is restricted to experiences from parents engaged in health care services. Triangulation with young people's narratives, including those that had not transitioned into adult services but re‐entered services in adulthood, both supported and challenged the typology. As family circumstances and attitudes to ADHD and health care services will vary, further research is needed to explore the fit of this typology with specific population groups such as families with lower socioeconomic status. Further study is also warranted to explore how a superfan role influences the illness‐related work undertaken and how health care systems could take on some of the identified work of the manager to better enable parents to move into the role of a roadie.

## Supporting information


**Table S1:** Supporting quotes.

## Data Availability

The data that supports the findings of this study are available in the supplementary material of this article. The full interview transcripts are available on request from the corresponding author. These data are not publicly available due to privacy or ethical restrictions.
