# Supplementary material for: Parenting roles for young people with attention‐deficit/hyperactivity disorder transitioning to adult services
Source: Dev Med Child Neurol. 2022 Jun 20;65(1):136–44. doi: 10.1111/dmcn.15320 (PMC10083944; doi:10.1111/dmcn.15320)
Supplement: Supplementary file 1 — Table S1: Supporting quotes. [file DMCN-65-136-s001.docx]

## Table S1: Supporting Quotes

| Parent as Manager | | |
| --- | --- | --- |
| Parental quotes | Essential nature (i) | Taking him to CBT [cognitive behavioural therapy], to CAMHS, arranging stuff. Slept with him until 7 just so he would sleep… I help him to wash, bathe and that sort of thing G4-S05-004  She won’t be moved [to adult services] or she wouldn’t have treatment if I weren’t involved. She would just so ‘no, it’s too much’… I have to do the lead role. As I say we discuss everything, medication or therapies or whatever G4-S05-015 |
|  | (no) Social network (ii) | He has amazing friends that stick by him through thick and thin. They were almost like his little conscience G4-S02-031  If I weren’t there I don’t know what she’d do, She doesn’t have close friends she can talk to… she doesn’t have a job, is dependent on me G4-S04-015 |
|  | Navigating services: skilled work (iii) | [Son] doesn’t fit into certain services/clinics so gets shunted over until they are acutely unwell G4-S05-004  I think we learnt quite early on that sometimes thinking that everything is being dealt with and they are busy and you are waiting your turn and they’ll get in touch, it’s not always the case… there have been times when I didn’t chase stuff up because I was trying to do the right thing and actually I should have chased it up because there had been an error G4-S04-046 |
|  | Managing the ADHD (iv) | I take [son] to all his appointments, I help him with his medications, I sit and I talk to him, try and explain certain strategies he could try… I know it’s very difficult for them because I know how hard it is for me to understand it, so I can just actually imagine how hard it is for them not knowing what’s really happening G4-S05-003  I have [the medication] locked away and I will give it to her morning and night… Yeah, I’ve had to run up to the school before “I ain’t taking my medication” G4-S04-015  He’s had Concerta in increasing doses… we have occasionally added methylphenidate in the evenings… to try and help him [with his homework] … We did try [Atomxetine] last year… It didn’t really help his hyperactivity very much and it did lower his mood… We are monitoring [his weight]… He doesn’t eat a good diet and will sometimes miss meals because of the anorexia Concerta gives him G4-S02-027 |
|  | Juggling ADHD management with other tasks (v) | It was such a job getting him on [Employment Support Allowance], I don’t want him to come off, we are going to be in such a mess… I can’t afford to keep him as I’m not working either. I really want him to work. It’s a vicious circle G4-S04-060  It wasn’t until 17 that we were at our wits end that I asked for private diagnosis… I am still fighting to get support. They wanted to discharge him and I think it’s too early G4-S03-012 |
|  | Expert carer required (vi) | It’s always me because no-one else will deal with him. I am the one who can calm him down G4-S01-018  I think taking meds will continue in the future… Some things I think she’s doing very, very well in and other things I think we’re going to have to keep an extra eye on that G4-S04-015 |
|  | Justifying role due to increased risks (vii) | Their impulsivity gets you and them in trouble. You are anxious, you can’t turn your back on them. They might be climbing out of a 3rd floor window G4-S02-027  People say he’s all right and physically he looks okay. But he can’t cross the road. Someone has to be with him when he goes to the toilet in the supermarket, because he’ll talk to anyone… It’s a worry him being kept safe. If given instructions he’ll do it but he needs supervision all the time… Sometimes he lives in his own world which is perfect for him… As they get older they mix with different people… he can’t go off medication, he’ll end up in trouble with police G4-S02-031 |
| Youth quotes | Mum is in control (viii) | There is a massive lack of funding in all mental health services. To actually get a diagnosis to start with is quite a big thing, even if that did take my mum pushing since I was 7 years old to get any kind of referral.  **Really?**  Yeah. It took years and years and years.  **What do you think made the difference, I don't know if you remember back then?**  It would have been my mum pushing. Because so many people I know have problems and then they've been turned away because they aren't at breaking point G2-S01-039  **Obviously your mum is here with you today but is it something that you think you could do yourself?**  Depending on what it is. If the medication, Mum has to sort it out because I’d probably just forget it.  **Yes, okay. Could you do the making appointments and coming to appointments on your own or does Mum always support you with that?**  Mum always supports me with that.  **Do you think you will get better at managing it yourself as you get older or do you think it’s always something you are going to need support with?**  Probably. It will probably be easier when I get older G2-S04-112 |
|  | Reliant on parent (also in adulthood) (ix) | I’m trying to learn a bit to be more independent because I don’t want him thinking I’m relying on my mum too much, because now I’m a mum myself, I’ve got to do it myself. So, it’s different now G2-S05-110  So now you are back in [service] and you are getting medication regularly and you are being seen regularly, do you feel confident in managing your own care, like managing your ADHD?  No, he does that, all of that.  So, you’ve got help from your partner for that?  He does everything. He had to leave his job to become my carer G3-S04-106  Would anything have made it easier for you?  No I just let me mum deal with it, she sorts me out.  And did she help you?  … I can only give you advice as much as I know and she looked after me my whole life, so she knows me best and she knows all my symptoms and any question you want to give her, she could tell you G3-S04-045 |
| Parent as Roadie | | |
| Parent quotes | Working towards independence (x) | We’re trying to get him independent because we’re not always going to be here G4-S02-031  I’ve come to the conclusion now or realisation that he’s never going to get rid of the ADHD. We don’t use it as a crutch for anything and I’ve told him that basically he needs to understand what his issues are and actually learn to deal with them G4-S01-086 |
|  | Standing back (xi) | If he goes to [town]… and the bus doesn’t turn up, he’s lost, he panics, and he phones me and I have to go find him… At home I have to say “right, it’s time for bed”. I shouldn’t have to, but I do because he’ll just sit there all night. So, there’s elements that we still have to manage but… He’s a young adult. We’re moving him into that ethos. He’s got a job, he’s at college, he’s learning independence… We’ve gone from me physically making him take his meds to him self-medicating… I said “if you don’t want to take them [son], that’s up to you. That’s your choice, but it’s your day you have got to live with, so if you find that you can’t do your day then you’ve got your own answer, haven’t you?... I’m helping him become an adult. I’m moving him secretly into his life G4-S01-018  I attend appointments with him… I try not to manage his time and routine. I put an appointment in his diary and then he needs to plan around it G4-S01-047 |
|  | Need for consistent support (xii) | I want him to have some help to understand how he can sort himself out and how he can control it himself… Driving, work, getting more qualifications is our next stage. So, consistency and someone to help manage his ADHD in the next few years would be helpful G4-S01-086  We’re going through that silly stage now where everybody thinks he’s a man and he’s cured G4-S02-031 |
|  | Maturation (xiii) | As soon as [son] had a valid voice and had the capacity to see the bigger picture he said he couldn’t see the benefit, only negatives. So [son] now makes own decision about treatment G4-S04-046  He didn’t like who he was and he didn’t want to be who he was… now he’s embracing it… it’ll always be a part of his life… He is accepting and just trying to do the best he can with what he’s got G4-S02-057 |
| Youth Quotes | Parent is my back-up (xiv) | I'm at that stage in life where I'm perfectly capable of looking after myself, I can cook for myself… I can clean… But because my brain works at 50,000 miles an hour, I find myself so mentally exhausted from existing that I wouldn't manage without her [mother] looking after me. It's not that I'm not capable it's just that I don't have the energy in my brain to even consider starting to do that sort of stuff. My mum has always been very involved. G2-S01-020  How confident do you feel in managing your own kind of care and needs?  Not brilliantly. I have anxiety issues as well… I do rely quite a lot on my mum for help with that… I’m not good at forethought and planning, and like with the dosage I was on, my mum was the one who made me aware that it didn’t work… I just wanted to sit on the 54 because I can’t be bothered… trying to deal with my GP… trying to deal with services, but I did need more than that. Yes, I’m not 100% confident in managing myself G2-S05-117 |
| Parents as Superfan | | |
| Parental quotes | Unconditional love (xv) | From fourteen to seventeen we fought to keep him in school. He wouldn’t take the meds, got in with the wrong crowd and self-medicated with cannabis. It came to a head when he hit his boss in a fit of rage… He finds it hard to stay in work… He doesn’t think before he speaks… his new job, there are random drug tests. If he declares his treatment, he’ll not be fit for work G4-S04-044 |
|  | Stand by your child (xvi) | Parents have to deal with the fallout and the flack. Behavioural problems are generally less with the drugs, but you still have to be very strict parents which takes it out of you a bit… Before you see anyone in mental health you are already stressed and negative with that child. You have an apparently badly behaved child and antagonism from other adults and parents. G4-S02-027  He panicked and nearly ripped hospital apart. I explained to the nurse what he needed and from that moment he was happy. So, I go with him [to appointments] G4-S01-018 |
|  | Fulfil their potential (xvii) | Although I didn’t want him on the drugs, I think I was thinking I wanted a magic answer just before his GCSEs so that he could actually produce GCSEs that reflected his ability G4-S04-049  I feel that when you have a child and you have that longing for their success in the future, you plan their life, that they’re going to go to school, that they’re going to get a good education, that they’re going to go to university, they’re going to get a good job, that rug has been pulled spectacularly out from underneath us G4-S04-049 |
|  | Finding your child’s niche (xviii) | There’s parents like myself, who know that if you don’t intervene, if you don’t work hard, that child will never achieve a lifestyle they dream about G4-S01-018  Because she’s quirky and loud she’s started getting into music photography… I think that would fit with who she is. She wouldn’t fit into an office environment G4-S01-040 |
|  | Support them in ‘feeling normal’ (xix) | [Daughter] has never wanted to be labelled… she was anti going anywhere, talking to anybody about it…. she knew she was different, she didn’t want to be reminded of the fact she was different, she just desperately wanted to be like everyone else G4-S03-008  He just doesn’t want to engage in anything because he just wants to be normal… details of condition got out at school, other pupils teased him… you keep it to yourself because you don’t want people to reject you because you are weird G4-S04-046 |
|  | Managing services: Hard work (xx) | Going to CAMHS is so daunting… As with all mental health services, there’s such a stigma attached... Having that weight of responsibility that your child has to access mental health services G4-S02-0006  I thought I can’t give my child a medication in order to try and make her fit the world, so I took her off it. I just wasn’t happy with it G4-S03-008 |
| Youth quotes | Crediting parents (xxi) | You mentioned your mum a little bit though, is your mum involved quite a lot?  Oh, yeah… my parents have been amazing through it all. So, part parents, part medication… If I didn’t have the parents that I did, I’m scared to imagine where I would’ve ended up because it’s not good G2-S05-096 |
| Parents not taking on the illness-related work | | |
| Youth Quotes | Clashing views on illness (and treatment) (xxii) | My dad tells me, ‘You don't need this’… He's from a completely different generation though… I understand where he's coming from, but I need to talk to someone on a regular basis G3-S05-067  My parents told the doctor that I was never to go back on the medication. So yes, I was told [by my parents] to take the IQ tablets and homeopathy medication that I didn’t really agree with and didn’t want to take G3-S03-077 |
|  | Negative Perceptions and exclusion (xxiii) | My mum will never say… I’m a disappointment to her – [but] when she talks to my aunty… some of the things that she says, she may not think it hurts me, but it hurts to hear my mum say these things G3-S05-067  Mother: if it wasn’t for the likes of me being a complete nuisance and going into college or going into school, and [other parent] as well, [child’s name] would probably struggle more, although you don’t like us doing it sometimes, do you?  I’ve got no choice. You are doing it anyway. You don’t speak to me for ages you just do it anyway.  Mum: No, we always speak to you first.  Even if I say ‘no’ you still do it G3-S07-055 |
